# Post-exercise Cold Water Immersion Effects on Physiological Adaptations to Resistance Training and the Underlying Mechanisms in Skeletal Muscle: A Narrative Review

**DOI:** 10.3389/fspor.2021.660291

**Published:** 2021-04-08

**Authors:** Aaron C. Petersen, Jackson J. Fyfe

**Affiliations:** ^1^Institute for Health and Sport, Victoria University, Melbourne, VIC, Australia; ^2^Deakin University, Institute for Physical Activity and Nutrition (IPAN), School of Exercise and Nutrition Science, Geelong, VIC, Australia

**Keywords:** cold-water immersion, resistance exercise, exercise performance, skeletal muscle, molecular responses, adaptation

## Abstract

Post-exercise cold-water immersion (CWI) is a popular recovery modality aimed at minimizing fatigue and hastening recovery following exercise. In this regard, CWI has been shown to be beneficial for accelerating post-exercise recovery of various parameters including muscle strength, muscle soreness, inflammation, muscle damage, and perceptions of fatigue. Improved recovery following an exercise session facilitated by CWI is thought to enhance the quality and training load of subsequent training sessions, thereby providing a greater training stimulus for long-term physiological adaptations. However, studies investigating the long-term effects of repeated post-exercise CWI instead suggest CWI may attenuate physiological adaptations to exercise training in a mode-specific manner. Specifically, there is evidence post-exercise CWI can attenuate improvements in physiological adaptations to resistance training, including aspects of maximal strength, power, and skeletal muscle hypertrophy, without negatively influencing endurance training adaptations. Several studies have investigated the effects of CWI on the molecular responses to resistance exercise in an attempt to identify the mechanisms by which CWI attenuates physiological adaptations to resistance training. Although evidence is limited, it appears that CWI attenuates the activation of anabolic signaling pathways and the increase in muscle protein synthesis following acute and chronic resistance exercise, which may mediate the negative effects of CWI on long-term resistance training adaptations. There are, however, a number of methodological factors that must be considered when interpreting evidence for the effects of post-exercise CWI on physiological adaptations to resistance training and the potential underlying mechanisms. This review outlines and critiques the available evidence on the effects of CWI on long-term resistance training adaptations and the underlying molecular mechanisms in skeletal muscle, and suggests potential directions for future research to further elucidate the effects of CWI on resistance training adaptations.

## Introduction

Cold water immersion (CWI) is a popular recovery strategy aimed at enhancing recovery from strenuous exercise. Typical CWI protocols involve the submersion of the limbs and/or torso for ~5–20 min in water cooled to temperatures of between ~8–15°C (Versey et al., 2013). Application of CWI usually occurs shortly after exercise cessation and may be performed either continuously [e.g., 1 bout of 15 min at 10°C (Fyfe et al., 2019)] or intermittently [e.g., 3 bouts of 4 min at ~12°C with 30 s between bouts (Frohlich et al., 2014)].

Application of CWI has been associated with a number of short-term benefits related to post-exercise recovery [as reviewed in Versey et al. (2013)], including a faster recovery of muscle strength (Skurvydas et al., 2006; Bailey et al., 2007; Vaile et al., 2008), muscle soreness (Bailey et al., 2007; Vaile et al., 2008; Ingram et al., 2009; Rowsell et al., 2011; Stanley et al., 2012), perception of fatigue (Parouty et al., 2010; Stacey et al., 2010; Rowsell et al., 2011; Stanley et al., 2012), and markers of inflammation (Montgomery et al., 2008; Peake et al., 2008; Stacey et al., 2010; Pournot et al., 2011) and muscle damage (Eston and Peters, 1999; Skurvydas et al., 2006) after strenuous exercise. However, evidence of the short-term benefits of CWI is equivocal, with some studies finding no influence of CWI on various aspects of post-exercise recovery including muscle strength (Paddon-Jones and Quigley, 1997; Goodall and Howatson, 2008; Howatson et al., 2009; Jakeman et al., 2009; Peiffer et al., 2009), muscle soreness (Paddon-Jones and Quigley, 1997; Sellwood et al., 2007; Howatson et al., 2009; Jakeman et al., 2009), and markers of muscle damage (Eston and Peters, 1999; Bailey et al., 2007; Goodall and Howatson, 2008; Jakeman et al., 2009) and inflammation (Montgomery et al., 2008; Peake et al., 2008). The potential short-term benefits of CWI are nevertheless thought to be primarily mediated by the local vasoconstriction and increased hydrostatic pressure attributed to the cold water temperature and depth associated with CWI, respectively (Wilcock et al., 2006). These factors are thought to exert various physiological effects, including decreased metabolic activity (Ihsan et al., 2013), altered hormonal responses (Earp et al., 2019), infiltration of immune cells (Lee et al., 2005), and reduced limb blood flow (Gregson et al., 2011; Mawhinney et al., 2013, 2017). Ultimately, these purported short-term benefits of CWI are theorized to enhance physiological adaptations to exercise training by improving the quantity and/or quality of subsequent training sessions (Barnett, 2006).

While post-exercise application of CWI can accelerate aspects of post-exercise recovery and enhance subsequent exercise performance, there is accumulating evidence that CWI can influence long-term physiological adaptations to exercise, and in a manner that is exercise mode-specific (Malta et al., 2020). For example, there is accumulating evidence that CWI can attenuate improvements in physiological adaptations to resistance training (including muscle hypertrophy and improvements in strength and power/rate of force development) (Roberts et al., 2015; Fyfe et al., 2019; Poppendieck et al., 2020), whereas CWI associated with endurance training does not appear to influence related adaptations including improvements in cycling time trial performance (either mean power or duration) or maximal aerobic power (Yamane et al., 2006; Halson et al., 2014; Broatch et al., 2017). Mechanistically, the mode-specific influence of CWI on physiological adaptations to exercise training is likely attributed to the short-term physiological effects of CWI on post-exercise molecular-level responses (in skeletal muscle in particular) that mediate physiological adaptations to exercise training.

The following sections will firstly summarize and critique the evidence for the influence of CWI on physiological adaptations to resistance training, including skeletal muscle hypertrophy and improvements in measures of maximal strength, strength endurance, and power/rate of force development, before discussing the potential molecular-level mechanisms in skeletal muscle underlying these effects. Finally, the limitations of current evidence, as well as potential directions for future research, are discussed.

## Influence of CWI on Physiological Adaptations to Resistance Training

Accumulating evidence suggests post-exercise CWI modulates physiological adaptations to exercise training in a mode-specific manner, with a negative influence on aspects of resistance training adaptations, but not on endurance training adaptations (Malta et al., 2020). Modes of exercise can be broadly defined as either endurance/aerobic training, consisting of relatively low-force muscle contractions performed for prolonged durations (such as running/cycling/swimming), or resistance/strength training, characterized by relatively high-force yet brief contractions performed intermittently. The principle of specificity in exercise training dictates that physiological responses, and in turn physiological adaptations, to exercise are highly specific to the mode of exercise performed. Resistance training is the most effective non-pharmacological intervention known to increase skeletal muscle mass and improve both the capacity (strength) and rate (power) of force production by skeletal muscle. For this reason, resistance training (particularly the associated improvements in force production ability) can aid performance enhancement in various athletic disciplines (Suchomel et al., 2016), and also attenuate declines in these parameters occurring across the lifespan that can impair functional ability and increase the risk of both morbidity and mortality (Maestroni et al., 2020).

Given the importance of physiological adaptations to resistance training for optimizing performance and health outcomes, factors that influence the magnitude of these adaptations have critical importance for maximizing the benefits of resistance training. Owing to the popularity of CWI as a post-exercise recovery technique, the potential influence of CWI on physiological adaptations to exercise training, including resistance training, has received increased attention in the literature. The following sections will describe the growing evidence that CWI can influence changes in various physiological adaptations to resistance training, including skeletal muscle hypertrophy, maximal strength, strength endurance, and aspects of power/rate of force development (RFD). While outside the scope of this narrative review, readers are instead referred elsewhere for discussion of the effects of post-exercise CWI on physiological adaptations to endurance training (Broatch et al., 2018; Malta et al., 2020). A summary of studies investigating the effects of CWI on physiological adaptations to resistance training is provided in Table 1.

**Table 1:** Summary of post-exercise cold-water immersion effects on physiological adaptations to resistance training.

**Study**	**Participants**			**Study design**	**Recovery intervention**	**Resistance training intervention**				**Main findings**
	**Sample size**	**Age**	**Resistance training status**			**Intervention length**	**Exercises trained**	**Frequency**	**Volume/intensity**	
Ohnishi et al. (2004)	16 (M)	20.1 ± 2.3 y	Not described	Within-subject/parallel group, repeated measures	CWI: 20 min at 10 ± 1°C CON: Passive sitting for 20 min	6 weeks	Handgrip exercise	3 × /week	3 × 8-RM	Muscle hypertrophy ↔ Forearm circumference for both control and CWI groups Maximal strength ↔ Isometric (handgrip) strength for both control and CWI groups Strength endurance ↑Number of handgrips (30% 1-RM until volitional fatigue) for both control and CWI groups • No difference between groups
Yamane et al. (2006)	11 (7 M, 4 F)	20.5 ± 0.8 y	Not described	Within-subject, repeated measures	CWI: 20 min at 10 ± 1°C CON: Non-immersion at 25 ± 1°C	4 weeks	Wrist flexion exercise	3 x/week	3 × 8-RM (2 min rest between sets)	Maximal strength ↑ Isometric (handgrip) strength for both control and CWI groups • Greater ↑ for control vs. CWI group • Strength endurance • ↑ Number of handgrips (30% 1-RM until volitional fatigue) for control group, but ↔ for CWI group
	16 (M)	20.7 ± 2.3 y	Not described	Parallel-group, repeated measures	CWI: 20 min at 10 ± 1°C CON: Non-immersion at 25 ± 1°C	4 weeks	Wrist flexion exercise	3 x/week	3 × 8-RM (2 min rest between sets)	Muscle hypertrophy ↔ Muscle thickness (wrist flexors, ultrasound) for both control and CWI groups Maximal strength ↔ Isometric (handgrip) strength for both control and CWI groups Strength endurance • ↑ Number of handgrips (30% 1-RM until volitional fatigue) for both control and CWI groups • No difference between groups
Frohlich et al. (2014)	17 (M)	23.5 ± 2.4 y	At least 6 months of resistance training experience (range 6 months to 5 years).	Within-subject, repeated measures	CWI: 3 * 4 min at 12 ± 1.5°C CON: Non-immersion at 20–23°C	5 weeks	Leg curl	2 x/week	3 × 8–12 repetitions (75–80% 1-RM)	Maximal strength ↑ Dynamic (both 1-RM and 12-RM leg curl) strength for both groups • Greater ↑ in 12-RM for control vs. CWI group • No difference in 1-RM between groups
Yamane et al. (2015)	14 (M)	20.2 ± 0.9 y	Recreationally active with no resistance training experience in past year.	Within-subject/parallel group, repeated measures	CWI: 20 min at 10 ± 1°C CON: Non-immersion at room temperature	6 weeks	Wrist flexion exercise	3 x/week	5 × 8 repetitions at 70–80% 1-RM)	Muscle hypertrophy ↑ Muscle thickness (wrist flexors, ultrasound) and forearm circumference for both control and CWI groups • Greater↑ in both measures for control vs. CWI • Maximal strength • ↑ Maximal isometric (wrist flexor) strength for control group, but ↔ for CWI group Strength endurance ↑ Number of handgrips (35% 1-RM until volitional fatigue) for both control and CWI groups • No difference between groups
Roberts et al. (2015)	21 (M)	21.2 ± 2.2 (CWI group) 21.3 ± 1.9 y (CON group)	At least 12 months experience with resistance training.	Parallel-group, repeated measures	CWI: 10 min at 10.1 ± 0.3°C CON: 10 min active recovery (cycling) at self-selected low intensity (~60 W)	12 weeks	Leg press Knee extension Knee flexion Walking lunges Plyometric exercises (drop jumps, slow eccentric squat jumps, split lunge jumps, countermovement box jumps)	2 x/week	3–6 × 8–12 RM (1 min rest between sets)	Muscle hypertrophy ↑ Muscle mass (quadriceps, MRI) for both control and CWI groups • Greater ↑ in for control vs. CWI group • ↑ Muscle fiber CSA (type II and combined type I + type II) for control group, but ↔ for CWI group • Maximal strength • ↑ Dynamic 1-RM (leg press and leg extension) strength for both control and CWI groups • ↑ Post-training values for control vs. CWI groups • ↑ Isometric (knee extensor, 70°) torque for control group, but ↔ for CWI group • ↑ Post-training values for control vs. CWI groups • ↔ Isokinetic (knee extensor, 90°/s) strength for both control and CWI groups Strength endurance ↑ Isokinetic work (knee extensors, contractions 1–25 of 50, 90°/s) for control group, but ↔ for CWI group • ↔ Isokinetic work (knee extensors, contractions 26–50 of 50, 90°/s) for either control or CWI groups • Power/RFD • ↑ Isometric RFD impulse (knee extensors, 70°) for both control and CWI groups • ↑ Post-training values for control vs. CWI groups
Fyfe et al. (2019)	16 (M)	25.0 ± 4.9 y	Recreationally-active, no resistance training experience in past 6 months	Parallel-group, repeated measures	CWI: 15 min at 10°C • CON: Non-immersion at 23°C	7 weeks	Back squat Barbell bench press Lat pulldown Walking lunges Shoulder press Bicep curl Tricep extension Lying leg raise (+ variants for each performed on alternate days)	3 x/week	3 × 12-RM or 20-RM (2 min recovery between sets)	Muscle hypertrophy ↑ Lean mass (total, lower-body and upper-body, DXA) for both control and CWI groups (combined) • No difference between groups • ↔ Muscle fiber (type I) CSA for both groups combined • Greater ↑ in muscle fiber (type II) CSA for the control vs. CWI groups • Maximal strength • ↑ Dynamic 1-RM (leg press and bench press) strength for both control and CWI groups (combined) • No difference between groups • Power/RFD • ↑ CMJ peak force for control group but ↔ for CWI group • Greater ↑ in for control vs. CWI group • ↔ Peak force during squat jump or ballistic push-up for both control and CWI groups (combined)
Poppendieck et al. (2020)	11 (9M, 2F)	25.3 ± 3.6 y	At least 6 months of resistance training experience (1–2 sessions per week).	Parallel-group, repeated measures	CWI: 10 min at 14–15°C	8 weeks	Leg press Leg curl Leg extension	3 x/week	3 × 10-RM (3 min recovery between sets)	Muscle hypertrophy ↑ Muscle thickness (vastus medialis, ultrasound) and thigh circumference for the control group, but ↔ for the CWI group • Small (*g* = 0.27) and large (*g* = 1.20) effects favoring the control vs. CWI group for leg circumference and muscle thickness, respectively • Maximal strength • ↔ Dynamic 1-RM (leg press) strength for both control and CWI groups • Power/RFD • ↔ CMJ height for both control and CWI groups

*1-RM, one-repetition maximum; CSA, cross-sectional area; CON, control; CWI, cold water immersion; RFD, rate of force development; DXA, dual x-ray absorptiometry; CMJ, countermovement jump; ↑, statistically significant (p < 0.05) increase with training, ↓ statistically significant (p < 0.05) decrease with training, ↔ no statistically significant (p > 0.05) change with training*.

### Skeletal Muscle Hypertrophy

Resistance training is a well-established strategy for increasing skeletal muscle mass—a process known as skeletal muscle hypertrophy (Haun et al., 2019). Before discussing current evidence for the influence of post-exercise CWI application on muscle hypertrophic responses to resistance training, there are several important conceptual and methodological factors related to the assessment of muscle hypertrophy worthy of consideration.

Skeletal muscle hypertrophy is a complex biological construct that may be assessed at different physiological levels (i.e., whole-body, macroscopic, microscopic, and molecular levels), and by using various measurement techniques each differing in aspects of validity, reliability, and specificity (Haun et al., 2019). Whole-body assessments typically measure changes in total or regional lean body/fat-free mass using methods such as Dual X-ray Absorptiometry (DXA), air displacement plethysmography (e.g., BodPod), or bioelectrical impedance analysis/spectroscopy (BIA/BIS). Macroscopic assessments of muscle hypertrophy typically assess changes in whole-muscle/limb size or cross-sectional area (CSA) *via* imaging techniques (such as MRI, CT, or ultrasound) or anthropometric (e.g., limb girth) measurements. Microscopic assessments of muscle hypertrophy assess changes in muscle fiber size and/or muscle fiber type by applying immunohistochemical techniques to skeletal muscle samples obtained *via* muscle biopsy. Less commonly applied in contemporary human exercise studies, molecular-level assessments of muscle hypertrophy involve the quantification of protein sub-fractions (e.g., myofibrillar or sarcoplasmic protein concentrations) within skeletal muscle samples obtained *via* muscle biopsy.

**Figure 1:**
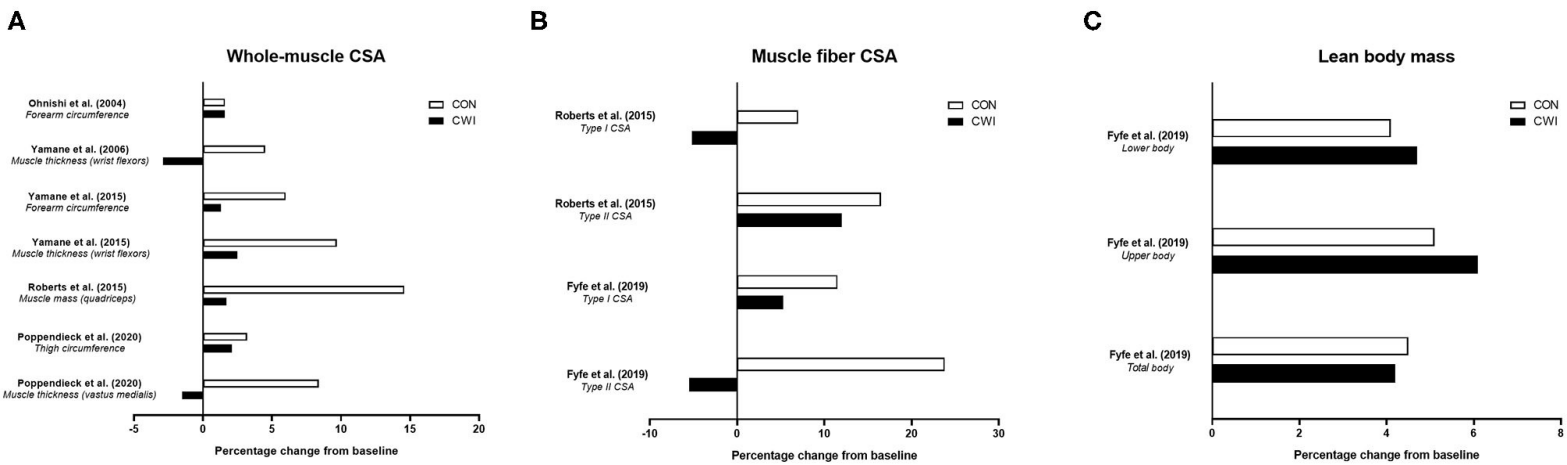
Summary of studies investigating the effects of post-exercise cold water immersion (CWI) on muscle hypertrophic adaptations to resistance training, including effects (shown as mean percentage changes from baseline to post-training) on measures of whole-muscle cross-sectional area (CSA; **A**), muscle fiber CSA **(B)**, and lean body mass **(C)**.

Human studies performed to date have investigated whether CWI influences skeletal muscle hypertrophic responses to resistance training at the whole-body (Fyfe et al., 2019), macroscopic (Ohnishi et al., 2004; Yamane et al., 2006, 2015; Roberts et al., 2015; Poppendieck et al., 2020), and microscopic (Roberts et al., 2015; Fyfe et al., 2019) levels (Figure 1). The findings of these studies have been mixed, with some suggesting CWI attenuates resistance training-induced increases in whole-muscle/limb size or cross-sectional area (CSA) (Roberts et al., 2015; Yamane et al., 2015; Poppendieck et al., 2020) and muscle fiber CSA (Roberts et al., 2015; Fyfe et al., 2019), while others have shown no influence of CWI on changes in either muscle/limb size or CSA (Ohnishi et al., 2004; Yamane et al., 2006) or total body or regional lean mass (assessed *via* DXA) (Fyfe et al., 2019) with resistance training.

Three studies (Roberts et al., 2015; Yamane et al., 2015; Poppendieck et al., 2020) have provided evidence for attenuated macroscopic-level (whole-muscle) hypertrophy following resistance training with CWI application. In the only study performed to date using a gold-standard assessment of muscle mass or CSA (MRI) (Roberts et al., 2015), post-exercise application of CWI (10 min at 10.1 ± 0.3°C) attenuated the increase in quadriceps muscle mass (~+15% for control vs. ~+2% for CWI) after 12 weeks of resistance training in young resistance-trained men. Two other studies in young, non-resistance trained males (Yamane et al., 2015) or resistance-trained males and females (Poppendieck et al., 2020) found CWI blunted the resistance training-induced increases in both forearm circumference and wrist flexor muscle thickness (Yamane et al., 2015) and in both thigh circumference and quadriceps (vastus medialis) muscle thickness (Poppendieck et al., 2020). The remaining studies that assessed macroscopic-level muscle hypertrophy found no influence of CWI on resistance training-induced changes in total or regional lean body mass (assessed *via* DXA) (Fyfe et al., 2019), wrist flexor muscle thickness (ultrasound) (Yamane et al., 2006), or forearm circumference (assessed anthropometrically) (Ohnishi et al., 2004) in young, non-resistance trained males.

While the majority of studies performed to date have assessed the influence of CWI on macroscopic-level muscle hypertrophy following resistance exercise, two studies (Roberts et al., 2015; Fyfe et al., 2019) have examined microscopic-level hypertrophic responses. Both studies showed that CWI attenuated the resistance training-induced increase in vastus lateralis type II muscle fiber area, with one study (Roberts et al., 2015) also suggesting that combined type I and type II muscle fiber areas (which may have been driven by the change in type II muscle fiber area) were enhanced by resistance training only with an active post-recovery (low-intensity cycling), but not with CWI.

To summarize, there is mixed evidence for the influence of CWI on indices of skeletal muscle hypertrophy, with three of six total studies showing attenuated whole-muscle hypertrophy of either the thigh (Roberts et al., 2015; Poppendieck et al., 2020) or wrist flexor (Yamane et al., 2015) musculature, and both of two available studies (Roberts et al., 2015; Fyfe et al., 2019) showing a negative influence of CWI on muscle fiber (specifically type II) hypertrophy. There is also no evidence that post-exercise CWI has beneficial effects on measures of skeletal muscle hypertrophy.

### Maximal Strength

Maximal strength is defined as the capacity of the neuromuscular system to produce force against an external resistance (Suchomel et al., 2016), and may be assessed using multiple methods including dynamic strength [involving concentric and/or eccentric actions, typically assessed as the one-repetition maximum (1-RM) load for a given exercise], isometric strength, or isokinetic strength. Improvements in maximal strength occur due to a combination of neural and morphological adaptations (Folland and Williams, 2007), with the relative contribution of these factors to strength gain with resistance training subject to ongoing debate (Loenneke et al., 2019; Taber et al., 2019). Post-exercise CWI application may theoretically impair strength development with resistance training by interfering with the morphological contributors (e.g., muscle hypertrophy) to improved strength, while the potential effects of CWI on neural adaptations to resistance training remain unclear.

**Figure 2:**
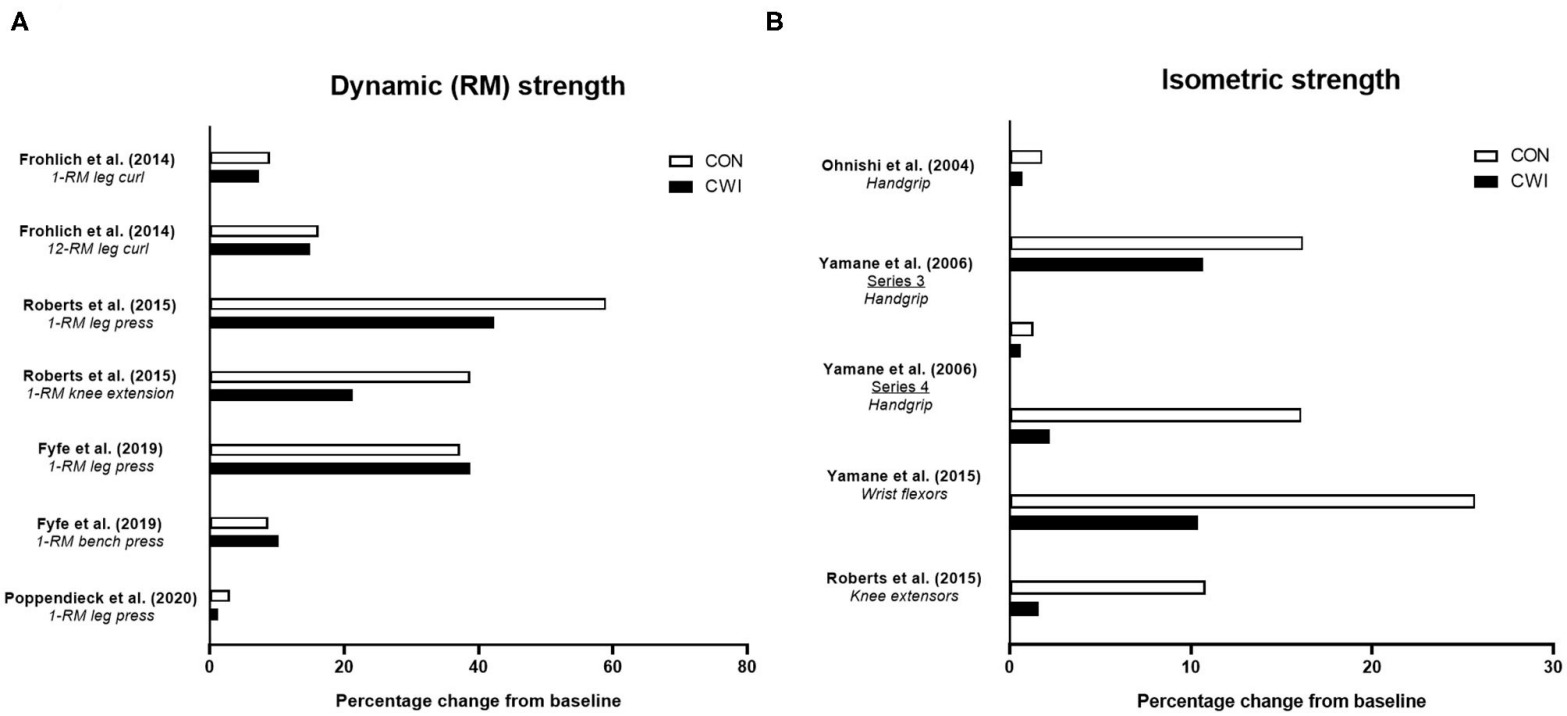
Summary of studies investigating the effects of post-exercise cold water immersion (CWI) on changes in maximal strength with resistance training, including effects (shown as mean percentage changes from baseline to post-training) on dynamic repetition-maximum (RM) strength **(A)** and isometric or isokinetic strength **(B)**. Adapted from Broatch et al. (2018) with permission.

To date, studies have shown mixed findings on the influence of CWI on improvements in various measures of strength with resistance training (Figure 2) (Ohnishi et al., 2004; Yamane et al., 2006, 2015; Frohlich et al., 2014; Roberts et al., 2015; Fyfe et al., 2019; Poppendieck et al., 2020). Four studies (Frohlich et al., 2014; Roberts et al., 2015; Fyfe et al., 2019; Poppendieck et al., 2020) have examined the influence of CWI on resistance training-induced changes in dynamic repetition-maximum (RM) strength and five studies (Ohnishi et al., 2004; Yamane et al., 2006, 2015; Frohlich et al., 2014; Roberts et al., 2015) have assessed isometric strength, while only one study (Roberts et al., 2015) has determined changes in isokinetic strength.

In the first study [of only three total studies (Frohlich et al., 2014; Roberts et al., 2015; Poppendieck et al., 2020)] to determine the influence of post-exercise CWI on dynamic RM strength gains with resistance training in participants with resistance training experience, Frohlich et al. (2014) assessed the influence of post-exercise intermittent CWI (3 bouts of 4 min at 12 ± 1.5°C) on dynamic (both 1-RM and 12-RM) leg curl strength gain after 5 weeks of resistance training in young males. The findings suggested post-exercise CWI application impaired the change in 12-RM strength (~+16% for control vs. ~+15% for CWI), while there was a tendency for greater 1-RM strength gain in the control group (~+9 vs. ~+7% for the CWI group) that was not statistically significant. In young resistance-trained males and females, Poppendieck et al. (2020) also found no influence of post-exercise CWI on 1-RM (leg press) strength gain, which did not improve in either the control or CWI group (~+3% vs. ~+1.5%, respectively) following 8 weeks of resistance training, although there was a small effect for less 1-RM strength gain in the CWI group. The findings of these studies were, however, contrasted by Roberts et al. (2015) who noted that alongside the attenuated muscle hypertrophy responses observed, post-exercise CWI application blunted the improvement in 1-RM leg press strength (~+59 vs. ~+42% for control and CWI, respectively), 1-RM knee extension strength (~+39 vs. ~+21%, respectively), and isometric knee extensor strength (~+26 vs. ~+10%, respectively) after 12 weeks of resistance training in young, resistance-trained males.

Taken together, these studies provide mixed evidence for attenuated dynamic, lower-body RM (1-RM or 12-RM) strength gain following resistance training with CWI application in resistance-trained individuals (Frohlich et al., 2014; Roberts et al., 2015; Poppendieck et al., 2020). More recently, findings from our laboratory in non-resistance-trained males (Fyfe et al., 2019) suggested CWI did not impair dynamic strength development of either the lower- (1-RM leg press) or upper-body (1-RM bench press) after 7 weeks of resistance training. The lack of negative influence of CWI on strength gain occurred despite CWI impairing vastus lateralis type II muscle fiber hypertrophy (but not total or regional lean body mass assessed *via* DXA), highlighting the potential disconnect between changes in measures of strength and muscle hypertrophy with resistance training (Loenneke et al., 2019).

Current evidence (Roberts et al., 2015; Fyfe et al., 2019) therefore provides mixed support for the notion that attenuated strength gain following resistance training with CWI application may be mediated by the negative effects of post-exercise CWI on skeletal muscle hypertrophy. We (Fyfe et al., 2019) theorized the discrepancies in findings on the influence of CWI on dynamic 1-RM strength gain between our study and Roberts et al. (2015) may have related to differences in task complexity of the strength measures chosen and the associated implications for the relative contribution of hypertrophic and non-hypertrophic mechanisms to strength gain. More complex motor tasks likely evoke a greater neural (i.e., non-hypertrophic) contribution to strength gain with resistance training (Rutherford and Jones, 1986), and neural adaptations may be less susceptible to interference from CWI compared with morphological adaptations (e.g., muscle hypertrophy). For this reason, it is possible that strength gain may be attenuated to a greater extent with CWI when assessed during less-complex movements (e.g., or isometric vs. dynamic exercises, or single-joint vs. multi-joint dynamic exercises) that likely involve a greater relative contribution of hypertrophic adaptations to strength gain. Nevertheless, findings on the influence of CWI on isometric strength assessed during less-complex movements has also been mixed. In contrast with the findings of Roberts et al. (2015) who observed a blunted improvement in isometric knee extensor strength following resistance training with CWI, Frohlich et al. (2014) found no influence of CWI on the improvement in isometric knee flexor strength with resistance training. Two other studies from Yamane and colleagues (Yamane et al., 2006, 2015) showed mixed effects of CWI on isometric wrist flexor strength with resistance training, with one study (Yamane et al., 2015) suggesting impaired isometric strength development with CWI, and the other (Yamane et al., 2006) showing no improvement in either group—a conclusion shared with earlier findings by Ohnishi et al. (2004). It is also possible that between-study differences in the resistance training status of the participants studied may explain the discrepant findings regarding the influence of post-exercise CWI on strength gain with resistance training. Since the relative magnitude of strength gain is larger in untrained vs. resistance-trained individuals, and is largely mediated by neural (i.e., non-hypertrophic) adaptations (Del Vecchio et al., 2019a), post-exercise CWI may therefore have less influence on strength gain in untrained populations. Nevertheless, further studies are needed to confirm whether resistance training status indeed influences the effects of post-exercise CWI on strength gain with resistance training.

In summary, only limited evidence exists on the influence of CWI on isokinetic strength development, with one study (Roberts et al., 2015) showing maximal isokinetic knee extension torque was not improved following resistance training combined with either CWI or control. There is mixed evidence on the influence of post-exercise CWI application on improvements in dynamic 1-RM and isometric strength with resistance training, with limited evidence on isokinetic strength gain. Only single studies have shown clear effects for blunted dynamic 1-RM (leg press) (Roberts et al., 2015) or 12-RM (leg curl) (Frohlich et al., 2014) strength gain with CWI, both in resistance-trained participants, and in isometric strength gain of the knee flexors (Roberts et al., 2015) or wrist flexors (Yamane et al., 2015) in those with and without resistance training experience, respectively. Although the findings of the available literature on the influence of CWI on strength development with resistance training are mixed, a recent meta-analysis (Malta et al., 2020) nevertheless concluded that post-exercise CWI attenuated the improvements in both dynamic (1-RM; ES = −0.50) and isometric (ES = −0.65) strength.

### Strength Endurance

Strength endurance (also known as local muscular endurance) describes the ability to withstand fatigue during sustained force production, which is underpinned by various physiological factors, including mitochondrial and capillary density, muscle fiber-type proportions, and muscle buffer capacity (Kraemer and Ratamess, 2004). Resistance training, particularly when sets are performed with lighter loads (e.g., ≥12–15-RM) for a prolonged duration and with minimal between-set recovery (e.g., ≤ 1 min), is a well-established strategy for improving strength endurance. Application of CWI could theoretically influence improvements in strength endurance by modulating changes in the aforementioned factors with resistance training (as discussed in section Effects of CWI on Molecular Responses to Resistance Training).

**Figure 3:**
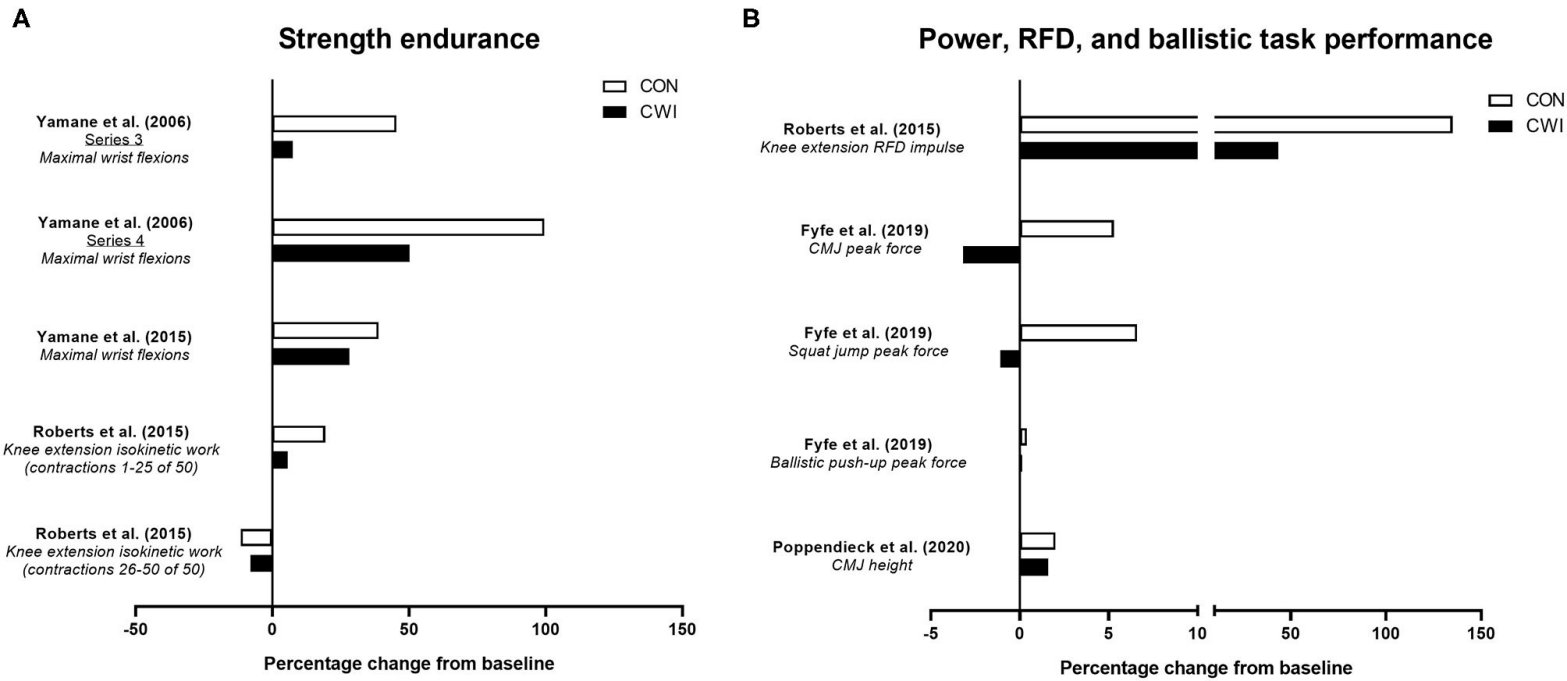
Summary of studies investigating the effects of post-exercise cold water immersion (CWI) on changes in strength endurance **(A)** and measures related to power, rate of force development (RFD) or ballistic task performance **(B)** with resistance training. Effects are shown as mean percentage changes from baseline to post-training.

Four studies have determined the influence of CWI on improvements in strength endurance of the wrist flexors (Ohnishi et al., 2004; Yamane et al., 2006, 2015) or knee extensors (Roberts et al., 2015) with resistance training (Figure 3A). In an initial study, Ohnishi et al. (2004) found strength endurance of the wrist flexors improved after 6 weeks of handgrip resistance training (3 × 8-RM) either with post-exercise CWI (20 min at ~10°C) of the forearm or with the control (no water immersion) group, but noted the improvement tended to be smaller in the CWI condition. Follow-up studies (Yamane et al., 2006, 2015) using an identical CWI protocol undertaken after similar RT protocols (or passive control) showed mixed effects of CWI on strength endurance. One study (Yamane et al., 2006) showed that in one cohort of participants, CWI blunted the improvement in wrist flexor strength endurance vs. the control group, while in a second cohort of participants, similar improvements in strength endurance occurred in both the CWI and control groups. A more recent study (Yamane et al., 2015) found that improvements in wrist flexor strength endurance occurred following both CWI and control, although there appeared to be less improvement following CWI. Although not a traditional measure of strength endurance *per se*, Roberts et al. (2015) found that isokinetic work completed over the first 25 of 50 total contractions was improved in the control group, but not in the CWI group, suggesting less improvement in aspects of strength endurance with CWI.

The limited available evidence therefore suggests CWI may attenuate improvements in strength endurance with resistance training, albeit when assessed during single-joint movements involving smaller muscle groups (i.e., wrist flexors). The physiological mechanisms for the negative effects of CWI on changes in strength endurance with resistance training remain unclear.

### Power, Rate of Force Development, and Ballistic Task Performance

The ability to produce force rapidly (variously described as mechanical power or rate of force development) is recognized as an important component of athletic performance (Cormie et al., 2011) and a key contributor to declines in functional capacity with aging (Foldvari et al., 2000). Neural adaptations are a key determinant of rate of force development (Del Vecchio et al., 2019b), with a smaller relative contribution from muscle morphological factors due to their association with maximal voluntary strength/torque (Maden-Wilkinson et al., 2021).

Resistance training is a well-established strategy for improving various aspects of power development (Cormie et al., 2011), and a limited number of studies have determined whether post-exercise CWI influences power-related adaptation to resistance training (Figure 3B). Roberts et al. (2015) were the first to show that power-related adaptations, specifically the improvement in isometric rate of force development of the knee extensors, was blunted (~+135% for control vs. ~+44% for CWI) after 12 weeks of resistance training with CWI application. While this suggests CWI can impair resistance training-induced improvements in rate of force development during simple, isometric movements, other studies have determined whether CWI influences performance improvements in rapid, dynamic movements such as the countermovement jump (CMJ) (Fyfe et al., 2019; Poppendieck et al., 2020), which may be more relevant to both athletic performance and activities of daily living. For example, we (Fyfe et al., 2019) showed the improvement in peak force during a CMJ (but not during a squat jump or ballistic push-up) with resistance training was impaired (~+5% for control vs. ~-3% for CWI) with post-exercise CWI, suggesting CWI application may compromise improvements in the ability to produce force during rapid, dynamic movements. The limited evidence on the influence of CWI application on CMJ performance outcomes with resistance training is however equivocal, with Poppendieck et al. (2020) finding no change in CMJ height after 8 weeks of resistance training (with or without CWI), although moderate negative effects of CWI were noted after a 3-week follow-up period.

Taken together, the limited available evidence suggests that improvements in the ability to produce force rapidly during either isometric or dynamic (CMJ) movements with resistance training may be compromised by post-exercise CWI application. Whether these effects are attributed to the influence of CWI on morphological and/or neural adaptations is, however, unclear.

## Effects of CWI on Molecular Responses to Resistance Training

**Figure 4:**
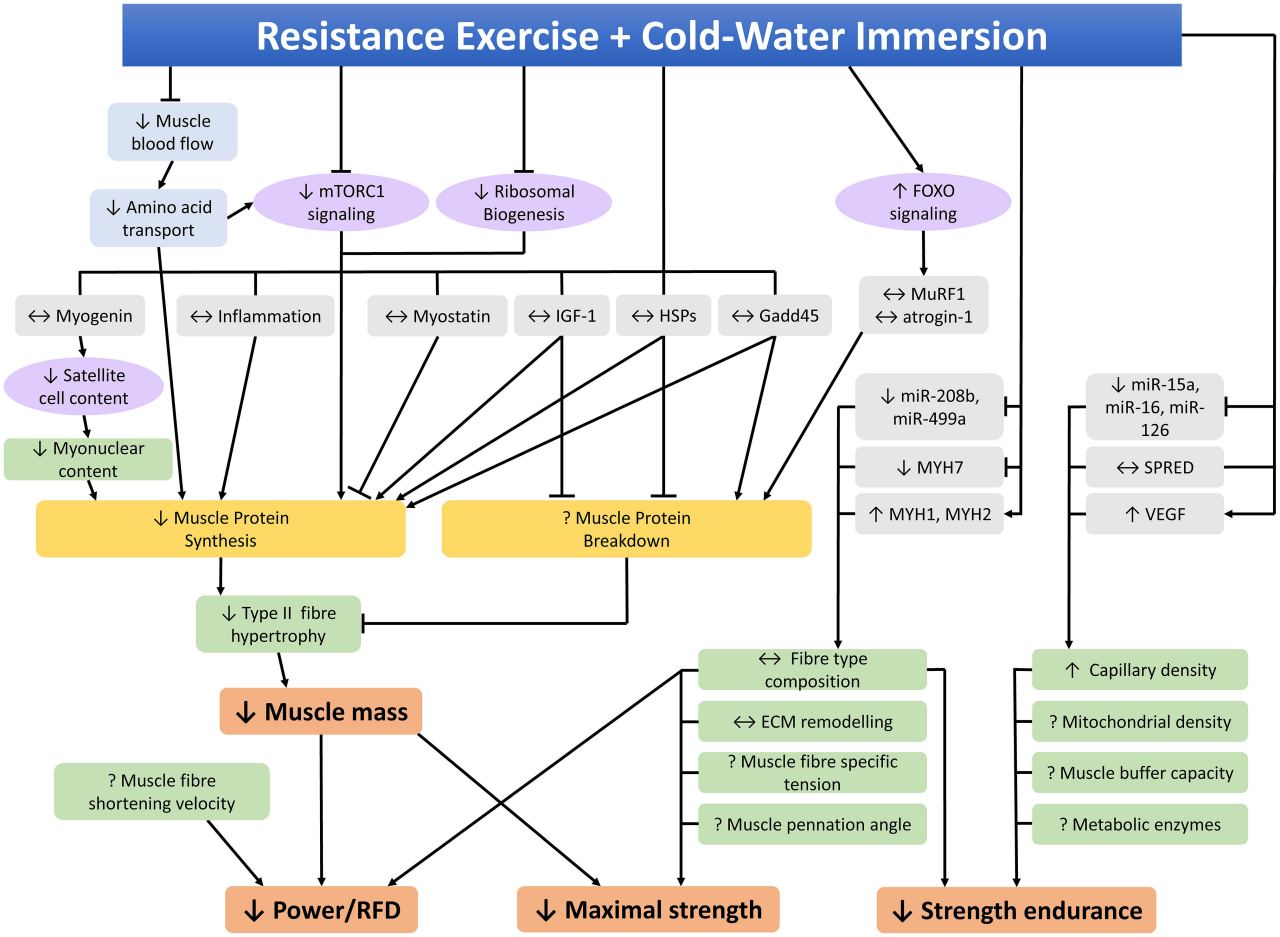
Molecular mechanisms within skeletal muscle that may contribute to the effects of post-exercise cold-water immersion on adaptations to resistance training. ↑, ↓, ↔ indicates increased, decreased, or unchanged response compared to the control condition, ? indicates that the effects of cold-water immersion on this variable have not been investigated, *RFD* rate of force development.

Several studies have investigated the effects of CWI on the molecular responses to resistance training to try to identify the mechanisms by which CWI attenuates phenotypic adaptations to resistance training. A summary of studies investigating the potential molecular mechanisms that may contribute to the effects of CWI on adaptations to resistance training in human skeletal muscle is provided in Table 2. An integrated summary of these molecular mechanisms demonstrating their interactions and potential links to performance outcomes is shown in Figure 4. The following section of the review discusses the effects of CWI on each of the mechanisms identified in Figure 4.

**Table 2:** Summary of post-exercise cold-water immersion effects on molecular responses to resistance exercise in human skeletal muscle.

	**Participants**			**Study design**	**Recovery intervention**	**Resistance training intervention**				**Muscle sampling times**	**Outcome measures (effects of CWI compared to control)**
**Study**	**Sample size (sex)**	**Age**	**Resistance training status**			**Intervention length**	**Exercises trained**	**Frequency**	**Volume/ intensity**		
Roberts et al. (2015)	21 (M)	21.2 ± 2.2 (CWI group) 21.3 ± 1.9 y (CON group)	At least 12 months experience with resistance training.	Parallel-group, repeated measures	CWI: 10 min at 10.1 ± 0.3°C CON: 10 min active recovery (cycling) at self-selected low intensity (~60 W)	12 weeks	Lower-body resistance exercises and plyometrics	2 x/week	3–6 × 8-12 RM (1 min rest between sets)	4–5 days pre-training 6–7 days post-training	↓ type II fiber CSA ↓ myonuclei per fiber
	9 (M)	22.1 ± 2.2	At least 12 months experience with resistance training.	Within-subject, crossover, repeated measures	CWI: 10 min at 10.1 ± 0.3°C CON: 10 min active recovery (cycling) at self-selected low intensity (~60 W)	N/A	Lower-body resistance exercises	Single exercise session	3–6 × 8–12 RM (1 min rest between sets)	Pre-exercise 2, 24, 48 h post-exercise	↓p70S6K protein at 48 h ↓ p-p70S6K^Thr421/Ser424^ at 2 and 24 h ↔ p-p70S6K^Thr389^ ↔ p-4E-BP1 ↓ rpS6 protein at 24 and 48 h ↔ p-rpS6^Ser240/244^ ↔ p-rpS6^Ser235/236^ ↓ PAX7^+^ satellite cells at 24 and 48 h ↓ NCAM^+^ satellite cells at 24 h ↔ ERK1/2 protein ↔ p-ERK1^Thr202/Tyr204^ ↔ p-ERK2^Thr185/Tyr187^ ↔ p-ERK1/2
Figueiredo et al. (2016)	9 (M)	22.1 ± 2.2	At least 12 months experience with resistance training.	Within-subject, crossover, repeated measures	CWI: 10 min at 10.1 ± 0.3°C CON: 10 min active recovery (cycling) at self-selected low intensity (~60 W)	N/A	Lower-body resistance exercises	Single exercise session	3–6 × 8–12 RM (1 min rest between sets)	Pre-exercise 2, 24, 48 h post-exercise	↓ p-p38^Thr180/Tyr182^ at 2 h (tendency *p* = 0.068) ↓ p-MNK1^Thr197^ at 2 h ↓ p-eIF4E^Ser209^ at 2h ↔ eIF4E protein ↓ Cyclin D1 protein at 2, 24, and 48 h ↔*Cyclin D1* mRNA ↓ p-Akt^Thr308^ at 48 h ↓ p-PRAS40^Thr246^ at 48 h ↓ rDNA transcription signaling (overall effect from several markers) ↓ pre rRNA expression (overall effect from several markers) ↓ rDNA transcription mRNA (overall effect from several markers)
Peake et al. (2017)	9 (M)	22.1 ± 2.2	At least 12 months experience with resistance training.	Within-subject, crossover, repeated measures	CWI: 10 min at 10.1 ± 0.3°C CON: 10 min active recovery (cycling) at self-selected low intensity (~60 W)	N/A	Lower-body resistance exercises	Single exercise session	3–6 × 8–12 RM (1 min rest between sets)	Pre-exercise 2, 24, 48 h post-exercise	↔ neutrophil (CD66b^+^) infiltration ↔ macrophage (CD68^+^) infiltration ↔ HSP70 cytosolic content ↔ HSP70 cytoskeletal content ↔αB-crystallin cytosolic content ↔αB-crystallin cytoskeletal content ↔αB-crystallin positive fibers ↔ macrophage (*MAC1, CD163*) mRNA ↔ cytokine and chemokine (*IL1β, TNF-α, IL6, CCL2, CCL4, CXCL2, IL8, LIF*) mRNA ↔*HSP70* mRNA
D'Souza et al. (2018)	21 (M)	21.2 ± 2.2 (CWI group) 21.3 ± 1.9 y (CON group)	At least 12 months experience with resistance training.	Parallel-group, repeated measures	CWI: 10 min at 10.1 ± 0.3°C CON: 10 min active recovery (cycling) at self-selected low intensity (~60 W)	12 weeks	Lower-body resistance exercises and plyometrics	2 x/week	3–6 × 8-12 RM (1 min rest between sets)	4–5 days pre-training 6–7 days post-training	↔ Fiber type % (type I, type IIa, type IIx and IIa/IIx) ↔ MyHCI, MyHCIIa protein ↓Δ*MYH7* mRNA (type I gene) ↑Δ*MYH2* (type IIa), Δ*MYH1* (type IIx) mRNA ↓ΔmiR-208b, ΔmiR-499a ↑ΔSox-6 ↑ capillaries per total fibers (tendency *p* = 0.051) ↑ capillaries around type II fibers ↔ capillaries around type I fibers ↑ΔVEGF protein ↔ΔSPRED-1 protein ↑Δ*VEGF1*, Δ*SPRED-1* mRNA ↓ΔmiR-15a, ΔmiR-16, ΔmiR-126
Fyfe et al. (2019)	16 (M)	20.9 ± 3.4 (CWI group) 25.0 ± 4.9 y (CON group)	Recreationally-active, no resistance training experience in past 6 months	Parallel-group, repeated measures	CWI: 15 min at 10°C CON: Non-immersion at 23°C	7 weeks	Whole-body resistance exercises	3 x/week	3 × 12–RM or 20-RM (2 min recovery between sets)	Pre-training (prior to first training session) Post-training (prior to last training session)	↔ type I CSA ↓ type II CSA ↔ p70S6K protein ↔ rpS6 protein ↔ 4E-BP1 protein ↑ FOXO1 protein ↔ FOXO3a protein ↔ MuRF-1 protein ↓ HSP27 protein ↓HSP72 protein ↔αB-crystallin protein
						N/A	Whole-body resistance exercises	Single session	3 × 12–RM or 20-RM (2 min recovery between sets)	Pre-exercise 1, 48 h post-exercise (performed during first (PRE) and last (POST) training sessions	↔ p-p70S6K^Thr389^c ↓ p-rps6^Ser235/236^ at POST 1 h and POST 48 h ↓ p-4E-BP1^Thr36/47^ at PRE 1 h ↓ p-FOXO1^Ser256^ at POST 1 h and POST 48 h ↔ p-FOXO3a^Ser253^ ↓ p-HSP27^Ser15^ at PRE 1 h ↔ p-HSP27^Ser82^ ↔ p- αB-crystallin ^Ser59^
Peake et al. (2020)	9 (M)	22.1 ± 2.2	At least 12 months experience with resistance training.	Within-subject, crossover, repeated measures	CWI: 10 min at 10.1 ± 0.3°C CON: 10 min active recovery (cycling) at self-selected low intensity (~60 W)	N/A	Lower-body resistance exercises	Single exercise session	3–6 × 8–12 RM (1 min rest between sets)	Pre-exercise 2, 24, 48 h post-exercise	↔ FOXO3a cytosolic expression ↔ FOXO3a nuclear expression ↔ Tenascin C protein ↔Δ*IGF-1 Ec*, Δ*IGF-1 Ea*, Δ*IGF-1 receptor* mRNA ↔Δ*Myogenin* mRNA ↔Δ*Gadd45a*, Δ*Gadd45b* mRNA ↔Δ*MuRF-1*, Δ*Atrogin-1* mRNA ↔Δ*Myostatin* mRNA ↔Δ*collagen type 1 alpha chain 1*, Δ*collagen type III alpha chain 1*, Δl*aminin subunit beta 1*, Δ*TIMP 1* mRNA
Fuchs et al. (2020)	12 (M)	21 ± 2	Recreationally active but not participating in structured resistance exercise	Within-subject, repeated measures	CWI (single leg): 20 min at 8°C CON (contralateral leg): 20 min at 30°C	2 weeks	Leg press, knee extension	3 x/week	4 × 10 RM (80% 1–RM)	2 h post-immersion following the first and last training sessions	↓daily myofibrillar protein FSR
						N/A	Leg press, knee extension	Single exercise session	4 × 10 RM (80% 1-RM)	0, 2, and 5 h post-immersion	↓L-[1-^13^C]-phenylalanine incorporation into Myofibrillar protein at 5 h ↓Myofibrillar protein FSR at 5 h ↔ mTOR^Ser2448^ ↔ p70S6K^Thr421/Ser424^ ↔ rpS6^Ser240/244^ ↔ rpS6^Ser235/236^ ↔ 4E-BP1^Thr37/46^ ↑p70S6K^Thr389^ at 0 but not 2 or 5 h ↔*FOXO1, MuRF1, atrogin-1* mRNA ↔*mTOR, p70S6K* mRNA ↔*GLUT4* mRNA ↔*IL-6* mRNA ↑*TNFα* mRNA at 0 h ↓SNAT2 protein at 0h ↓CD98 protein at 2 and 5 h

*Δ, change from pre- to post-training; 1-RM, one repetition maximum; 4E-BP1, eukaryotic translation initiation factor 4E-binding protein 1; Akt, protein kinase B; CCL2, Monocyte chemotactic protein 1; CCL4, Macrophage inflammatory protein 1β; CD163, cluster of differentiation 163; CON, control group or condition; CSA, cross-sectional area; CWI, cold-water immersion; CXCL2, Macrophage inflammatory protein 2α; eIF4E, eukaryotic translation initiation factor 4E; FOXO, forkhead box O; FSR, fractional synthesis rate; Gadd45, growth arrest and DNA damage-inducible protein 45; HSP, heat-shock protein; IGF-1, insulin-like growth factor 1; IL, interleukin; LIF, Leukemia inhibitory factor; MAC1, macrophage-1 antigen; miR, microRNA; MNK1, mitogen-activated protein kinase–interacting serine/threonine-protein kinase 1; MuRF-1, muscle-specific ring finger 1; MyHC, myosin heavy chain; NCAM, neural cell adhesion molecule; PAX7, paired box 7; p38, mitogen-activated protein kinase; PRAS40, protein-rich AKT1 substrate 1; p70S6K, ribosomal protein S6 kinase beta-1; rDNA, ribosomal deoxyribonucleic; rpS6, ribosomal protein S6; rRNA, ribosomal ribonucleic acid; SOX6, SRY-box 6; SPRED, sprouty-related EVH1 domain-containing protein; TIMP, tissue inhibitor of metallopeptidase; TNF-α, tumor necrosis factor alpha; VEGF, vascular endothelial growth factor; ↑, significantly greater than CON (p < 0.05); ↓, significantly less than CON (p < 0.05); ↔, not significantly different than CON (p > 0.05)*.

### Anabolic Responses

#### Protein Synthesis

As discussed previously (section Skeletal Muscle Hypertrophy), CWI can attenuate measures of muscle hypertrophy in response to resistance training (Roberts et al., 2015; Yamane et al., 2015; Fyfe et al., 2019; Poppendieck et al., 2020). Muscle hypertrophy in response to resistance training is driven primarily through transient increases in muscle protein synthesis (Biolo et al., 1997) and decreases in protein breakdown (Phillips et al., 1997), which suggests CWI may alter rates of protein synthesis and/or breakdown in response to resistance exercise.

Only one study to date has investigated the effects of CWI on muscle protein synthesis (Fuchs et al., 2020), demonstrating that 20 min of CWI (single-leg immersion in 8°C) attenuated myofibrillar protein synthesis rate by ~20% in the 5 h recovery period following an acute resistance exercise bout compared to the contralateral leg, which was immersed in 30°C water. These authors also investigated the chronic effects of post-exercise CWI on myofibrillar protein synthesis by applying D_2_O tracer methodology during a 2-week resistance training program consisting of seven lower-body training sessions. Single-leg immersion for 20 min in 8°C water after each training session reduced daily myofibrillar protein synthesis by 12% compared to the contralateral leg (Fuchs et al., 2020). Based on the limited available evidence, it therefore appears that CWI impairs the synthesis of muscle proteins in response to acute and chronic resistance exercise. Presumably, reduced muscle protein synthesis is a major contributor to the potential impairments in resistance training-induced muscle growth with CWI application; however, since changes in muscle size were not measured concurrently with muscle protein synthesis rates (Fuchs et al., 2020), this cannot be directly inferred. Future studies, which include concurrent measurement of protein synthesis and muscle size following resistance training with CWI application are needed to resolve this.

#### Anabolic Signaling

Transient increases in muscle protein synthesis in response to resistance exercise are primarily regulated by the mechanistic target of rapamycin complex 1 (mTORC1) signaling pathway, which controls protein translation by the ribosome (Bodine et al., 2001; Drummond et al., 2009; Goodman et al., 2011). Any effects of CWI on resistance exercise-induced muscle protein synthesis or hypertrophy may therefore be due to altered mTORC1 signaling. Three studies have investigated CWI effects on mTORC1 signaling, with conflicting findings. Roberts et al. (2015) reported that CWI blunted the post-exercise phosphorylation of p70S6K^Thr421/Ser424^ and there was a non-significant trend toward reduced phosphorylation of p70S6K^Thr389^, however there was no effect on several other markers of mTORC1 signaling, including phosphorylation of 4E-BP1 (as assessed by the mobility shift appearance of the γ isoform), rpS6^Ser240/244^, or rpS6^Ser235/236^. In contrast, we observed that CWI attenuated phosphorylation of rpS6^Ser235/236^ and 4E-BP1^Thr36/47^, but had no effect on p70S6K^Thr389^ phosphorylation (Fyfe et al., 2019). Despite impairing myofibrillar protein synthesis, CWI had no effect on phosphorylation of mTOR^Ser2448^, p70S6K^Thr421/Ser424^, rpS6^Ser235/236^, rpS6^Ser240/244^, or 4E-BP1^Thr36/47^ (Fuchs et al., 2020). Paradoxically, phosphorylation of p70S6K^Thr389^ was elevated in the CWI leg compared to the control leg immediately after immersion, suggesting elevated protein synthesis, however this effect had dissipated by 2 and 5 h after immersion (Fuchs et al., 2020). Overall, although the evidence is sparse and inconsistent, there may be some effects of CWI on resistance exercise-induced mTORC1 signaling. The absence of more robust effects suggests that other mechanisms are also likely to contribute to the CWI-induced inhibition of muscle protein synthesis and hypertrophy in response to resistance exercise.

#### Ribosome Biogenesis

Rates of protein translation, and thus protein synthesis, during periods of chronic resistance training depend not only on activation of translation by existing ribosomes but also on the capacity for protein translation, which is dependent on ribosomal content. As such, ribosomal biogenesis is likely to be important for muscle hypertrophy, as indicated during muscle overload in rodents (Goodman et al., 2011). Evidence suggests it may also be involved in regulating protein synthesis and muscle hypertrophy in response to resistance exercise in humans (Figueiredo et al., 2015; Fyfe et al., 2018). To date, only one study has investigated the effects of CWI on ribosomal biogenesis responses to resistance exercise. In that study, CWI following an acute resistance exercise bout attenuated signal transduction pathways and transcription of key genes involved in ribosome biogenesis, however it had no effect on the content of mature ribosomal RNA (rRNA) components, such as 28S, 18S, and 5.8S rRNA (Figueiredo et al., 2016). Nonetheless, the impairment of ribosomal biogenesis signaling and transcription suggests that increases in ribosomal content may be attenuated by CWI during chronic resistance training, however whether this occurs has yet to be investigated.

#### Satellite Cells

Satellite cells are involved in muscle regeneration following injury, however there is debate whether they are involved in resistance exercise-induced muscle hypertrophy. For example, depletion of satellite cells had no effect on muscle growth during short-term muscle overload in mice (McCarthy et al., 2011), but did reduce hypertrophy during more prolonged overload (Fry et al., 2014). In human models of resistance exercise, muscle growth was greatest in participants with the highest pre-training satellite cell population (Petrella et al., 2008) and muscle hypertrophy was accompanied by increased satellite cell content (Snijders et al., 2016). Thus, there is growing evidence that satellite cells do play a role in resistance-exercise-induced muscle hypertrophy in humans. Although evidence is limited, CWI appears to inhibit the satellite cell response to resistance exercise. The upregulation of paired box protein (Pax7) positive satellite cells, a marker of satellite cell abundance, following a single resistance exercise bout was completely blocked by CWI (Roberts et al., 2015). In the same study, the post-exercise increase in neural cell adhesion molecule (NCAM) positive satellite cells appeared to be delayed by CWI (Roberts et al., 2015). These responses are consistent with the observed satellite cell response to chronic resistance training, as the increase in type II muscle fiber myonuclear content was blocked by CWI following 12 weeks of lower body resistance training (Roberts et al., 2015). However, the mRNA expression of myogenin, which promotes differentiation of satellite cells into myonuclei (Asfour et al., 2018), was not altered by CWI following acute resistance exercise (Peake et al., 2020). These observations suggest the reduction in myonuclear content caused by CWI is due to impaired satellite cell proliferation and not differentiation.

#### Insulin-Like Growth Factor-1

Insulin-like growth factor-1 (IGF-1) is expressed systemically and locally within skeletal muscle. It is involved in promoting muscle hypertrophy, regeneration, and satellite cell proliferation and differentiation as well as inhibiting muscle protein degradation (Yoshida and Delafontaine, 2020). The mRNA expression of the IGF-1 receptor and the IGF-1 isoforms IGF-1Ea and IGF-1Ec in skeletal muscle were not different between the CWI and control groups following a resistance exercise bout (Peake et al., 2020), suggesting that CWI does not impair muscle hypertrophy *via* this pathway.

#### Mechanisms Contributing to Impaired Anabolic Responses

Reduced skeletal muscle blood flow and nutrient delivery to the muscle may contribute to the impaired anabolic response caused by CWI during recovery from resistance exercise. Several studies have shown that CWI reduces skeletal muscle blood flow (Gregson et al., 2011; Mawhinney et al., 2013) and muscle blood flow is positively related to increased rates of muscle protein synthesis (Fujita et al., 2006; Timmerman et al., 2010). The gene expression of some markers of amino acid transport was reduced in skeletal muscle by CWI following resistance exercise (Fuchs et al., 2020), which is consistent with reduced blood flow and would further reduce the availability of amino acids for muscle protein synthesis.

Another mechanism by which CWI may attenuate post-exercise anabolism is *via* its effects on inflammation. The inflammatory response is important for muscle repair following injury (Grisbrook et al., 2013; Urso, 2013) and appears to be at least partially involved in the post-exercise increase of muscle protein synthesis (Trappe et al., 2002). Although cold exposure is commonly cited as reducing post-exercise inflammation, much of the evidence to support this comes from animal models of muscle injury or human eccentric exercise models, which are not representative of typical resistance exercise due to the much greater muscle damage they induce. Indeed, studies investigating the inflammatory or immune cell response to resistance exercise show either no effect (Gonzalez et al., 2014a,b; Fragala et al., 2015; Jajtner et al., 2015; Yamane et al., 2015; Peake et al., 2017; Fuchs et al., 2020) or a potentiated response (Roberts et al., 2014; Jajtner et al., 2015; Fuchs et al., 2020) due to CWI, with only one study reporting a decreased response (Earp et al., 2019). Most studies have investigated the effects of CWI on systemic inflammation. Five studies showed unchanged levels of inflammation (Gonzalez et al., 2014a,b; Fragala et al., 2015; Jajtner et al., 2015; Yamane et al., 2015), two showed increased levels (Roberts et al., 2014; Jajtner et al., 2015) and one study observed decreased inflammation (Earp et al., 2019). Only two studies have investigated CWI effects on intramuscular inflammation. Similar to systemic inflammation, markers of intramuscular inflammation were either not affected (Peake et al., 2017; Fuchs et al., 2020) or were increased by CWI (Fuchs et al., 2020), thus indicating no clear difference between the effects of CWI on systemic and intramuscular inflammation. The majority of evidence therefore indicates that CWI effects on inflammation are unlikely to contribute to the attenuated anabolic response to resistance exercise.

### Catabolic Responses

#### Protein Breakdown

In addition to muscle protein synthesis, rates of muscle protein breakdown could influence net protein balance and therefore changes in muscle mass over time. To date, the effects of CWI on rates of muscle protein breakdown following resistance exercise have not been investigated, therefore it is currently unknown whether elevated muscle protein breakdown contributes to the impaired muscle hypertrophy observed in some studies following repeated post-exercise CWI. Although the effects of CWI on rates of muscle protein breakdown following resistance training have not been directly measured, some studies have investigated the molecular mechanisms that regulate muscle protein breakdown.

#### Ubiquitin Proteasome Pathway

Skeletal muscle protein breakdown is primarily controlled by the ubiquitin proteasome pathway (Goll et al., 2008). Key components of the ubiquitin proteasome pathway include the Forkhead Box O (FOXO) family of transcription factors, which are responsible for the regulation of numerous atrophy-related genes including the E3 ubiquitin ligases atrogin-1 and MuRF-1 (Milan et al., 2015). Atrogin-1 and MuRF-1 bind ubiquitin molecules to specific substrates, which includes myofibrillar proteins, thus targeting the ubiquitinated substrate for degradation by the 26S proteasome (Bodine and Baehr, 2014). CWI had no effect on the gene expression of several markers of the ubiquitin proteasome pathway, including FOXO1, MuRF-1, and atrogin-1 following an acute bout of resistance exercise (Fuchs et al., 2020; Peake et al., 2020). At the protein level, CWI after a single resistance exercise session had no effect on levels of FOXO3a within the cytosol or nucleus (Peake et al., 2020) or on phosphorylation of FOXO1^Ser256^ or FOXO3a^Ser253^ (Fyfe et al., 2019). The long-term effects of repeated CWI following a period of resistance training on markers of the ubiquitin proteasome pathway have only been investigated in one study. We observed that repeated CWI during 7-weeks of resistance training increased protein content of FOXO1 but had no effect on FOXO3a or MuRF-1 (Fyfe et al., 2019). Phosphorylation of FOXO1^Ser256^ at 1 and 48 h after the first resistance training session was not different between the control and the CWI groups. However, phosphorylation of FOXO1^Ser256^ after the last session of a 7-week resistance training program increased to a greater extent in the control compared to the CWI group (Fyfe et al., 2019). This indicates that repeated post-exercise CWI exposures may influence the molecular response to an acute post-exercise CWI exposure, which is consistent with the altered acute molecular responses observed before and after a period of exercise training (Wilkinson et al., 2008; Vissing et al., 2013). Since phosphorylation of FOXO1 at Ser256 reduces its DNA binding activity (Wang et al., 2016), the reduced phosphorylation caused by chronic CWI may promote higher rates of protein breakdown, while post-exercise phosphorylation of FOXO3a^Ser253^ was not altered by chronic CWI (Fyfe et al., 2019).

#### Myostatin

Myostatin is a negative regulator of muscle growth, which is typically downregulated for 24–48 h following resistance exercise (Hulmi et al., 2007; Louis et al., 2007). CWI may prolong the downregulation of myostatin mRNA following a resistance exercise bout, as it appeared to be reduced at 24 and 48 h post-exercise in the CWI group, whereas it was not different from pre-exercise in the control group (Peake et al., 2020). However, due to large variability within the results, there was no statistically significant difference from pre-exercise in either group, nor were there significant differences between groups.

#### Growth Arrest and DNA Damage-Inducible 45

Growth arrest and DNA damage-inducible 45 protein (Gadd45) is upregulated in response to anabolic stimuli, such as synergist ablation-induced overload (Carson et al., 2002) and resistance exercise (Peake et al., 2020) and also in response to catabolic stimuli, such as fasting and skeletal muscle denervation or immobilization (Ebert et al., 2012; Bongers et al., 2013), suggesting that it plays a role in muscle protein turnover or remodeling. The effects of CWI on Gadd45 have only been investigated in one study, whereby a single bout of resistance exercise increased the mRNA expression of Gadd45a and Gadd45b, however this response was not altered by CWI (Peake et al., 2020).

In summary, the available evidence indicates that CWI attenuates muscle hypertrophy in response to resistance exercise *via* a reduction in muscle protein synthesis, which appears to be driven by multiple factors, including blunted mTORC1 signaling, ribosomal biogenesis, myonuclear content, and muscle amino acid transport. Some, albeit very limited, evidence suggests that increased protein breakdown may also contribute to the reduced muscle hypertrophy caused by CWI, although this may only occur following repeated CWI exposures. Additional studies, which concurrently measure muscle mass and muscle protein synthesis or protein breakdown following resistance training with post-exercise CWI are required to determine whether CWI impairs muscle growth *via* altered muscle protein synthesis and/or muscle protein breakdown.

### Skeletal Muscle Remodeling

Muscular adaptations that contribute to increased strength following a period of resistance training involve not only muscle hypertrophy, but also skeletal muscle remodeling, which includes increased muscle fiber specific tension (Pansarasa et al., 2009), altered muscle fiber pennation angle (Folland and Williams, 2007), preferential hypertrophy of type II muscle fibers (Folland and Williams, 2007), and enhanced lateral transfer of force between the sarcomere and extracellular matrix (Erskine et al., 2011). Resistance training-induced increases in power/RFD may also involve altered fiber type, increased muscle fiber shortening velocity and other morphological factors (Pansarasa et al., 2009; Schiaffino and Reggiani, 2011; Maden-Wilkinson et al., 2021), whereas increased strength endurance may result from fiber type shifts, increased mitochondrial density, enhanced capillary density, improved muscle buffer capacity, and altered metabolic enzymes.

To date, the effects of CWI on only a few of the above-mentioned mechanisms has been investigated.

#### Extracellular Matrix Remodeling

The ECM is a scaffold of collagens and proteins that has multiple roles within skeletal muscle, one of which is the lateral transfer of force from the sarcomeres to the muscle connective tissue (Csapo et al., 2020). Increased lateral force transfer has been proposed as a mechanism of increased muscle specific tension following resistance training (Erskine et al., 2011). An acute bout of resistance exercise upregulates the mRNA expression of several ECM-related genes, including collagen type I alpha chain 1, collagen type III alpha chain 1, laminin, and tissue inhibitor of metallopeptidase 1, however the expression levels were not altered by CWI (Peake et al., 2020). Tenascin-C is upregulated in response to muscle contractions and is thought to be involved in ECM remodeling (Mackey and Kjaer, 2017). The protein content of tenascin-c was upregulated 24 h after a single resistance exercise session, however this was not altered by CWI. This suggests that CWI does not alter ECM remodeling in response to resistance exercise, however future studies investigating chronic effects of CWI on ECM proteins are warranted to confirm this.

#### Muscle Fiber Type Composition

In addition to muscle hypertrophy, a shifting of muscle fiber type composition is another classic adaptation to resistance training (Staron et al., 1990). Muscle fiber type shifts with exercise training typically manifest as conversions between fast-twitch type IIx and type IIa fibers (Staron et al., 1990, 1994), with conflicting observations of switching between type I and type II fiber types (Adams et al., 1993; Paddon-Jones et al., 2001). These muscle fiber type shifts are thought to promote a shift toward a more fatigue-resistant skeletal muscle phenotype.

Since muscle fiber type is a key determinant of its contractile properties (Schiaffino and Reggiani, 2011), potential shifts in muscle fiber type composition with CWI may influence changes in performance outcomes with resistance training, such as improvements in strength, power/rate of force development, and strength endurance.

There is indirect evidence suggesting cold exposure may promote a shift toward a faster muscle phenotype, with divers exposed to prolonged habitual CWI showing higher proportions of type IIx muscle fibers vs. physically-active controls (Bae et al., 2003), while type I to type II fiber type shifts were observed in rat soleus muscle following CWI (Walters and Constable, 1993; Lee et al., 2004).

Using data from a previous investigation (Roberts et al., 2015), only a single study (D'Souza et al., 2018) has determined whether post-exercise CWI influences muscle fiber type composition shifts with resistance training. The findings suggested that CWI did not alter the shifts in muscle fiber type composition measured *via* histological staining for myosin heavy chains (reduced percentage of type IIx fibers and increased percentage of type IIa fibers) seen with the control condition (active recovery). These findings were confirmed by Western blot analysis of MyHCI and MyHCIIa protein content, which did not differ between CWI and control. Interestingly, additional analyses of MYH7 (type I gene), MYH1 (type IIx gene), and MYH2 (type IIa gene) mRNA expression, as well as expression of two microRNAs thought to be involved in regulation of muscle fiber type (miR-208b and miR499a) were consistent with a CWI-induced type II muscle fiber shift (D'Souza et al., 2018). This raises the possibility that CWI effects on muscle fiber type may occur following a longer period.

#### Fiber Type-Specific Hypertrophy

In addition to altered muscle fiber type composition, fiber type-specific changes in muscle fiber size also occur following resistance training. Type II fibers appear to hypertrophy to a greater extent than type I fibers following resistance training (Thorstensson et al., 1976; Dons et al., 1979; Houston et al., 1983; Fyfe et al., 2019). There is also some evidence that type II muscle fibers have a higher specific tension and shortening velocity than type I fibers (D'Antona et al., 2006; Pansarasa et al., 2009). These two factors combined may therefore contribute to increased strength and power/RFD after a period of resistance training. No studies have yet investigated the effects of CWI on muscle fiber specific tension or shortening velocity, so it is unknown whether CWI-induced impairments in these variables may contribute to the reduced strength and power caused by CWI. As discussed in section Skeletal Muscle Hypertrophy, CWI attenuated the resistance training-induced increase in type II muscle fiber area (Roberts et al., 2015; Fyfe et al., 2019). This suggests that the smaller resistance training-induced gains in strength caused by CWI may be partially attributable to impaired growth of type II fibers. However, although changes in type I fiber cross-sectional area were not reported, the increase in type I and II fiber combined area was attenuated by CWI (Roberts et al., 2015). Thus, it is possible that CWI also blunts type I fiber hypertrophy. This may not have been detected in our study (Fyfe et al., 2019) as the 7-week training intervention was possibly too short to induce a substantial increase in type I fiber area.

### Cell Stress Response

The heat shock family of proteins are well-known for their roles in protection from cellular stress (Lindquist, 1986). Heat shock proteins are important for cellular homeostasis and protein preservation and degradation (Noble et al., 2008) and play key roles in several processes involved in adaptation to exercise. For example, HSP72 regulates processes involved in protein synthesis and degradation (Ku et al., 1995; Zhou and Thompson, 1997; Beere et al., 2000; Senf et al., 2008; Dokladny et al., 2015). The small HSPs, HSP27 and αB-crystallin, inhibit protein degradation pathways (Dodd et al., 2009; Vasconsuelo et al., 2010; Adhikari et al., 2011) and bind to cytoskeletal and myofibrillar proteins following muscle-damaging exercise, where they are thought to stabilize disrupted elements and assist in regeneration and remodeling (Koh and Escobedo, 2004; Paulsen et al., 2007). CWI following a single bout of resistance exercise had no effect on HSP72 mRNA expression or levels of HSP72 protein within cytosolic or cytoskeletal fractions (Peake et al., 2017), however HSP72 protein content was decreased by repeated CWI following 7 weeks of resistance training, whereas the control group was unchanged (Fyfe et al., 2019). CWI had no effect on the cytosolic or cytoskeletal content of αB-crystallin, the number of αB-crystallin positive fibers, or phosphorylation of αB-crystallin^Ser59^ following a resistance exercise session (Peake et al., 2017; Fyfe et al., 2019). Nor did CWI alter the increase in αB-crystallin protein content following resistance training (Fyfe et al., 2019). CWI amplified the increase in HSP27^Ser15^ phosphorylation following the first but not the last training session of a 7-week resistance training program, however it had no effects on the resistance exercise-induced phosphorylation of HSP27^Ser82^ (Fyfe et al., 2019). Following 7 weeks of resistance training HSP27 protein content increased to a greater extent in the control group compared to the CWI group. These combined results indicate that some components of the cellular stress response following resistance exercise are altered by CWI, which may contribute to the impaired adaptive responses observed.

### Angiogenesis

Angiogenesis, which describes the formation of new blood vessels, has been shown to occur in response to resistance training (Cocks et al., 2014; Verdijk et al., 2016; D'Souza et al., 2018; Holloway et al., 2018), however whether this is a proactive adaptation that facilitates muscle hypertrophy or a reactive adaptation in response to exercise-induced metabolic stressors and/or muscle fiber hypertrophy-driven changes in perfusion is unclear (Holloway et al., 2018). Following 12 weeks of resistance training, the number of capillaries per muscle fiber, the capillary-to-fiber perimeter exchange index, and capillary density increased in the CWI group but not in the control group (D'Souza et al., 2018). The authors also investigated some of the molecular mechanisms regulating angiogenesis and found that CWI increased the mRNA expression of the pro-angiogenic factor VEGF1 and the anti-angiogenic factor SPRED-1 compared to control (D'Souza et al., 2018). At the protein level, VEGF1 but not SPRED-1 content was higher in the CWI compared to the control group (D'Souza et al., 2018). The expression of several microRNAs that inhibit VEGF1 (miR-15a and miR-16) and SPRED-1 (miR-126) was also investigated, with each of these being upregulated in control and downregulated in CWI (D'Souza et al., 2018). Although this is the only study to investigate the effects of CWI on angiogenic responses to resistance training, the results indicate that CWI induces a predominantly pro-angiogenic environment. Since muscle fiber hypertrophy appears to be blunted by CWI, this suggests that the pro-angiogenic environment may occur in response to possible CWI-induced alterations in metabolic stressors or reduced muscle perfusion. Longer duration resistance training studies may be needed to determine whether the favorable effects of CWI on angiogenic signaling eventually translate to increased capillarization which may then enable muscle fiber hypertrophy.

## Limitations and Future Directions

While there appears to be little evidence for beneficial effects of CWI on physiological adaptation and molecular responses to resistance training, there are a number of limitations and additional considerations when interpreting the available evidence.

### Effectiveness of the Resistance Training Intervention

To determine whether CWI application influences physiological adaptations to resistance training, it is necessary to compare changes in training outcomes with post-exercise CWI to a control condition. The comparison in training-induced responses in the CWI and control conditions are therefore critical for drawing conclusions on whether CWI influences responses to resistance training alone. There are a number of examples in the literature whereby changes in outcome measures, including dynamic 1-RM strength (Poppendieck et al., 2020), isometric strength (Ohnishi et al., 2004; Yamane et al., 2006), isokinetic strength (Roberts et al., 2015), CMJ height (Poppendieck et al., 2020), or peak force during a squat jump or ballistic push-up (Fyfe et al., 2019), were not evident with resistance training when combined with neither post-exercise CWI or a control condition. It must be considered, however, that it may not be possible to demonstrate whether CWI had an influence on physiological adaptations if the resistance training intervention was itself (i.e., the control condition) not sufficient to elicit substantial changes in these outcomes. It is therefore likely that the effectiveness of a given resistance training intervention for eliciting physiological adaptations can influence interpretation of whether post-exercise CWI indeed modulates these responses.

Since the effectiveness of any exercise training intervention for eliciting physiological adaptations is dependent on a multitude of factors, including the specifics of the training intervention itself and characteristics of the participant cohort (e.g., age, sex, genetics, training status, nutritional status, among others), these factors must be considered when interpreting evidence for the influence of CWI on physiological adaptations to exercise training.

### Training Status of Participants

The majority of studies investigating whether CWI influences physiological adaptations to resistance training have been conducted in participants with limited or no resistance training experience. Indeed, only three studies (Frohlich et al., 2014; Roberts et al., 2015; Poppendieck et al., 2020) have been undertaken in participants with some degree of resistance training experience. The resistance training experience of participants likely has implications for both the ability of relatively short-term resistance training interventions to induce substantial changes in outcome measures (and in turn detect any potential influence of CWI on these outcomes), as well as the applicability of study findings to athletic populations.

The principle of diminishing returns suggests the magnitude of physiological adaptations to exercise training are reduced in trained compared with untrained individuals. It is therefore possible that detecting any potential effect of CWI on resistance training adaptations is more challenging in trained vs. untrained individuals. For example, one (Poppendieck et al., 2020) of the three studies (Frohlich et al., 2014; Roberts et al., 2015; Poppendieck et al., 2020) performed in resistance-trained individuals found dynamic 1-RM (leg press) strength did not improve with resistance training (either with CWI application or control), which precludes the possibility of detecting any potential influence of post-exercise CWI. Nevertheless, studies in trained individuals are essential to ensure the relevance of study findings to athletic populations, while longer-term training interventions may be necessary to induce the substantial changes in outcome measures required to understand the potential effects of CWI on training responses in these populations. Given the time course of resistance training adaptations, whereby neural adaptations are considered to largely mediate short-term improvements in muscle force characteristics (Del Vecchio et al., 2019a), with morphological adaptations traditionally considered to occur at a slower rate (Folland and Williams, 2007), the length of the resistance training interventions used by studies may also influence conclusions on the effects of CWI on muscle hypertrophy and aspects of strength or power development. Since the potential negative influence of post-exercise CWI on changes in aspects of muscle function (i.e., strength and power/RFD) with resistance training are thought to be mediated *via* the effects of CWI on muscle hypertrophy, rather than neural adaptations *per se*, it is possible that short-term resistance training interventions, particularly in non-resistance trained individuals, may therefore be less likely to find negative effects of CWI on these responses. To date, studies have employed resistance training interventions of between 4 and 12 weeks in duration (Ohnishi et al., 2004; Yamane et al., 2006, 2015; Frohlich et al., 2014; Roberts et al., 2015; Fyfe et al., 2019; Poppendieck et al., 2020). It is likely that longer interventions may be required, particularly in resistance-trained populations, to detect substantial changes in aspects of strength and/or power/RFD and particularly muscle hypertrophy, to determine whether CWI influences these adaptations relative to resistance training alone.

### Measures Used to Assess Muscle Hypertrophy Outcomes

Even if a given resistance training intervention is sufficient to elicit physiological adaptations including skeletal muscle hypertrophy and improvements in strength and/or power-related measures, the tools used to assess these responses can also influence the ability of a given study to detect these changes. The assessment of skeletal muscle hypertrophy is particularly challenging, due not only to conceptual issues when defining muscle hypertrophy as a biological construct, but also because of the multitude of tools available to assess indices of muscle hypertrophy at multiple physiological levels (Haun et al., 2019). While imaging techniques including MRI and CT are considered gold-standard for assessing changes in whole-muscle size/CSA, only a single study (Roberts et al., 2015) has used these methodologies to assess the influence of CWI on hypertrophic responses to resistance training.

It is worth noting that one (Fyfe et al., 2019) of the two studies (Roberts et al., 2015; Fyfe et al., 2019) that have assessed hypertrophic responses at both macroscopic and microscopic levels following resistance training with or without CWI showed mixed findings depending on the particular measure of hypertrophy used. Specifically, CWI application impaired the resistance training-induced increase in type II muscle fiber CSA of the vastus lateralis (~+12% for control vs. ~+5% for CWI) but did not affect improvements in lean mass assessed *via* DXA (Fyfe et al., 2019). It should be considered, however, that while DXA-derived assessments of lean mass are highly correlated (*r* = 0.85–0.94) with gold-standard assessments of whole-muscle size (e.g., MRI and CT) when measured at a single time point (Levine et al., 2000; Maden-Wilkinson et al., 2013), DXA may be less sensitive (*r* = 0.33 vs. CT) for detecting changes in indices of skeletal muscle hypertrophy following a period of resistance training (Delmonico et al., 2008). It is therefore unclear whether similar discrepancies between muscle hypertrophy outcomes assessed *via* muscle biopsy (muscle fiber CSA) and DXA (lean body mass) may have occurred if another gold-standard measurement of macroscopic-level muscle hypertrophy (e.g., MRI, CT, or ultrasound) was undertaken. Nevertheless, such discrepancies in muscle hypertrophy outcomes assessed at different physiological levels (e.g., macroscopic vs. microscopic) is consistent with other findings in response to resistance training alone (Narici et al., 1996; Aagaard et al., 2001; Esmarck et al., 2001). For example, several studies have shown greater resistance training-induced changes in vastus lateralis muscle CSA when assessed at the muscle fiber vs. whole-muscle levels (Narici et al., 1996; Aagaard et al., 2001; Esmarck et al., 2001). These observations further highlight the complexities of assessing hypertrophic responses in human skeletal muscle, and in turn, interpretation of the potential effects of CWI on resistance training-induced muscle growth.

Additional studies assessing muscle hypertrophy following resistance training with or without CWI at multiple physiological levels (e.g., whole-body, macroscopic, and microscopic) concurrently, and particularly using gold-standard measures (such as MRI or CT) are required to improve current understanding of the influence of CWI on muscle hypertrophy with resistance training.

### Measures Used to Assess Strength Outcomes

As when assessing the influence of CWI on skeletal muscle hypertrophic responses to resistance training, there are various considerations when interpreting changes in maximal strength with resistance training, and therefore the potential influence of CWI on these responses. Because strength is a highly task-specific phenomenon (Morrissey et al., 1995), the magnitude of strength gain following a resistance training intervention is influenced by various factors, including the complexity of the movement patterns performed within the training intervention, and the degree of similarity (in terms of movement patterns, range of motion, lifting velocity, and intensity/loads used) between the resistance training exercises and the methods used to assess changes in strength (Buckner et al., 2017). For these reasons, the potential influence of the type of resistance training performed on the magnitude of strength gain (which is also influenced by participant characteristics), as well as the specificity of methods used to assess changes in strength, should be considered when interpreting the effects of CWI (vs. a control condition) on strength development with resistance training.

The specificity of strength gains with resistance training has important implications for the measures used to detect changes in strength with resistance training. In some cases, it is possible the measure of strength chosen could influence (e.g., underestimate) changes in strength with resistance training, and in turn, compromise the ability to detect potential effects of CWI on resistance training-induced strength gain. In some studies investigating the effects of CWI on resistance training adaptations, the measure of strength used was somewhat inconsistent with the modality of resistance training employed. For example, the studies by Yamane et al. (2006, 2015) and Ohnishi et al. (2004) employed dynamic resistance training of the wrist flexors, but assessed changes in isometric handgrip strength as the sole strength outcome. It is therefore possible that strength gains may have been greater in magnitude if a dynamic strength measure was instead assessed, which may have increased the possibility of detecting any impairments to strength gain that may have been caused by post-exercise CWI.

Future studies should therefore include multiple measures of strength (e.g., dynamic RM, isometric, and/or isokinetic strength) (Buckner et al., 2017) assessed during tasks that replicate those in the resistance training intervention to best capture the potential influence of post-exercise CWI application on strength development with resistance training.

### Resistance Training Interventions Used

There are a number of limitations regarding the resistance training interventions used in existing studies, which likely influence their applicability to real-world scenarios. For example, some studies have used resistance training interventions that incorporate single exercises that target only smaller muscle groups during single-joint movements (Ohnishi et al., 2004; Yamane et al., 2006, 2015; Frohlich et al., 2014), with only three studies (Roberts et al., 2015; Fyfe et al., 2019; Poppendieck et al., 2020) incorporating exercises targeting larger muscle groups during multiple dynamic, multi-joint movements commonly found in resistance training interventions in athletic settings. Additionally, only a single study has employed a whole-body resistance training intervention (Fyfe et al., 2019), which may be necessary to determine whether CWI exerts local or systemic effects on physiological adaptations to resistance training (e.g., whether lower-body CWI influences upper-body muscle hypertrophy or strength/power), and to mimic common resistance training practices.

A further limitation regarding the relevance for elite athletes of studies performed to date is the relatively low training volumes and frequencies employed in those studies. Athletes typically train at least five times per week and often at considerably higher frequencies (Smith, 2003). At higher training frequencies, optimizing recovery between sessions is likely of greater importance to enhance the quality of subsequent training sessions, and presumably increase the stimulus for physiological adaptation. Up to now, CWI studies have used training frequencies of only two or three sessions per week, with at least 1 day of recovery between sessions. These relatively low training frequencies may allow adequate recovery between training sessions and therefore reduce any potential recovery-enhancing benefits of CWI that may be more important with higher training frequencies.

### Potential Sex-Specific Effects

Very few studies performed to date have involved female participants. The exceptions to this include Poppendieck et al. (2020), in which two of the 11 participants were females and Yamane et al. (2006), in which four of 27 participants were females. In both cases, male and female data were not presented or analyzed seperately, precluding any insight into whether sex-specific effects of CWI on resistance training outcomes occurred. Females typically have greater subcutaneous fat thickness and a higher surface area to body mass ratio than males (Kruschitz et al., 2013), which alters the level of cooling (Petrofsky and Laymon, 2009; Castellani and Young, 2016) and may therefore influence the effects of post-exercise CWI. Given the potential for sex-specific effects of CWI on adaptations to resistance training and the implications of this for a large proportion of athletes, further research in this area is warranted.

### Potential Acclimation Effects

Repeated or prolonged cold exposure results in acclimation, which alters the thermoregulatory and metabolic responses to cold (Castellani and Young, 2016). As a result, the effects of CWI may be altered following prolonged use. In support of this hypothesis, we observed different responses for phosphorylation of HSP27^Ser15^, FOXO1^Ser256^, and rpS6^Ser235/236^ in the CWI group following the last session of a 7-week resistance training program compared to the first session (Fyfe et al., 2019). However, whether acclimation to CWI changes its effects on resistance training-induced performance adaptations has not been investigated. Given the complexity of the various adaptations that occur with cold acclimation it is difficult to predict whether the effects of CWI would be attenuated or exacerbated with repeated CWI exposure.

## Conclusions

Post-exercise CWI is a widely-used recovery modality among athletes. Nonetheless, there are relatively few studies investigating the effects of repeated CWI on adaptations to exercise, especially resistance exercise. Although post-exercise CWI may enhance short-term recovery following resistance exercise, current evidence suggests CWI has either nil or detrimental effects on physiological adaptations to resistance training, including muscle hypertrophy and measures of maximal strength, strength endurance, and power/RFD, as well as the molecular responses that underpin adaptation to resistance training in skeletal muscle. Importantly, no studies have shown benefits of CWI on resistance training adaptations. As such, there is currently no evidence to support the use of post-exercise CWI during periods of resistance training. It is important to note, however, that given the lack of available evidence and its associated limitations, there may be many potential circumstances whereby CWI application following resistance training could be beneficial, such as in females, in chronically-trained and elite athletes, during periods of high-frequency training, or in cold-acclimated individuals. Further research is required to determine the effects of post-exercise CWI on physiological adaptations resistance training in these circumstances, and to address the additional methodological limitations of previous studies.

## Author Contributions

AP and JF contributed to the writing and editing of the article and approved the submitted version.

## Conflict of Interest

The authors declare that the research was conducted in the absence of any commercial or financial relationships that could be construed as a potential conflict of interest.
